# [^11^C]Choline PET/CT in therapy response assessment of a neoadjuvant therapy in locally advanced and high risk prostate cancer before radical prostatectomy

**DOI:** 10.18632/oncotarget.11653

**Published:** 2016-08-27

**Authors:** Sarah M. Schwarzenböck, Anna Knieling, Michael Souvatzoglou, Jens Kurth, Katja Steiger, Matthias Eiber, Irene Esposito, Margitta Retz, Hubert Kübler, Jürgen E. Gschwend, Markus Schwaiger, Bernd J. Krause, Mark Thalgott

**Affiliations:** ^1^ Department of Nuclear Medicine, Klinikum Rechts der Isar, Technische Universität München, 81675 Munich, Germany; ^2^ Department of Nuclear Medicine, Rostock University Medical Centre, 18057 Rostock, Germany; ^3^ Department of Nuclear Medicine, Ulm University, 89081 Ulm, Germany; ^4^ Institute of Pathology, Klinikum Rechts der Isar, Technische Universität München, 81675 Munich, Germany; ^5^ Institute of Pathology, Heinrich-Heine University of Düsseldorf, 40225 Düsseldorf, Germany; ^6^ Department of Urology, Klinikum Rechts der Isar, Technische Universität München, 81675 Munich, Germany

**Keywords:** prostate cancer, therapy response assessment, neoadjuvant therapy, choline, PET/CT

## Abstract

**Purpose:**

Recent studies have shown promising results of neoadjuvant therapy in prostate cancer (PC). The aim of this study was to evaluate the potential of [^11^C]Choline PET/CT in therapy response monitoring after combined neoadjuvant docetaxel chemotherapy and complete androgen blockade in locally advanced and high risk PC patients.

**Results:**

In [^11^C]Choline PET/CT there was a significant decrease of SUV_max_ and SUV_mean_ (*p* = 0.004, each), prostate volume (*p* = 0.005) and PSA value (*p* = 0.003) after combined neoadjuvant therapy. MRI showed a significant prostate and tumor volume reduction (*p* = 0.003 and 0.005, respectively). Number of apoptotic cells was significantly higher in prostatectomy specimens of the therapy group compared to pretherapeutic biopsies and the control group (*p* = 0.02 and 0.003, respectively).

**Methods:**

11 patients received two [^11^C]Choline PET/CT and MRI scans before and after combined neoadjuvant therapy followed by radical prostatectomy and pelvic lymph node dissection. [^11^C]Choline uptake, prostate and tumor volume, PSA value (before/after neoadjuvant therapy) and apoptosis (of pretherapeutic biopsy/posttherapeutic prostatectomy specimens of the therapy group and prostatectomy specimens of a matched control group without neoadjuvant therapy) were assessed and tested for differences and correlation using SPSS.

**Conclusions:**

The results showing a decrease in choline uptake after combined neoadjuvant therapy (paralleled by regressive and apoptotic changes in histopathology) confirm the potential of [^11^C]Choline PET/CT to monitor effects of neoadjuvant therapy in locally advanced and high risk PC patients. Further studies are recommended to evaluate its use during the course of neoadjuvant therapy for early response assessment.

## INTRODUCTION

Prostate cancer is the most common malignancy in men worldwide and the second most common cause of cancer-related deaths in men [1]. Up to 40% of prostate cancer patients present with locally advanced stages at initial diagnosis. Those patients have a high risk of biochemical recurrence after radical prostatectomy (RPE) [2, 3]. Although neoadjuvant treatment is not generally recommended by urological guidelines, there is a high interest in perioperative treatment modalities such as neoadjuvant therapeutic concepts including the use of solely androgen deprivation therapy or docetaxel monotherapy as well as a combination of both in order to improve the surgical outcome in patients with locally advanced and high risk prostate cancer. Several phase II studies have shown the feasibility of local tumor downstaging with a tumor volume reduction as well as reduction of PSA values after neoadjuvant androgen deprivation and/or docetaxel therapy; however, improvement of survival or induction of relevant complete pathological remission could not be shown [4–10]. Recently, Thalgott et al. 2014 [11] prospectively evaluated a presurgical short-term combination therapy (complete androgen blockade and docetaxel therapy) in 30 locally advanced and high risk prostate cancer patients before RPE. Morphological therapy response (namely changes in prostate volume and tumor stage) after neoadjuvant therapy was assessed using magnetic resonance imaging (MRI). However, besides morphological changes, such as a decrease in size (as detected by MRI), metabolic changes might occur during neoadjuvant therapy. These metabolic changes can be detected using functional imaging modalities, such as positron emission tomography/computed tomography (PET/CT), which has been used successfully in the preclinical as well as clinical setting for therapy response assessment in different tumor entities [12–14]. In metastasized castration refractory prostate cancer controversial results have been shown by Ceci et al. 2015 and Schwarzenböck et al. 2016 on the use of [^11^C]Choline PET/CT for therapy response assessment of docetaxel therapy [15, 16]. Only little data are available on the use of [^11^C]Choline PET/CT in monitoring response to neoadjuvant therapy in primary prostate cancer [17, 18].

### The aim of this study was to evaluate the potential of [^11^C]Choline PET/CT

Imaging in therapy response monitoring by the use of choline signal modulation after neoadjuvant therapy in patients with locally advanced and high risk prostate cancer before radical prostatectomy. We histopathologically determined regressive changes and apoptosis in pretherapeutic biopsy and posttherapeutic prostatectomy specimens of the therapy group and in prostatectomy specimens of a control group (without neoadjuvant therapy). Correlation between changes in choline signal, CT ad MRI derived prostate and tumor volume reduction, PSA decrease and apoptosis was analyzed.

## RESULTS

### Histopathological analysis and apoptotic rate

Histopathologically, prostatectomy specimens after neoadjuvant combination therapy showed an average of 42% (range 15–80%) therapy associated regressive changes. Neoplastic glands mostly showed an atrophic appearance with collapsed lumina, acellular clefts containing pyknotic cancer cells or mucinous pools. Additionally, in some cases, high numbers of tumor cells with an abundant xanthomatous cytoplasm or with a distinct cytoplasmic vacuolization were observed.

As expected, in pretherapeutic biopsy specimens of the therapy group as well as in radical prostatectomy specimens of patients without neoadjuvant therapy (control group), no regressive changes were detected (see Table 1).

**Table 1:** PET/CT derived pre- and posttherapeutic parameters as well as regressive changes and apoptosis of pretherapeutic biopsy specimens and posttherapeutic prostatectomy specimens of the therapy group and prostatectomy specimens of the control group

Parameters	Pretherapeutic	Posttherapeutic	Decrease in %	*p*-value
SUVmean				
median (mean)	3.45 (3.43)	2.28 (2.36)	31.3 (30.4)	0.004
range	2.84–3.87	1.67–4.07	26.4–56.9	
SUVmax				
median (mean)	7.13 (7.50)	3.57 (3.68)	52.1 (47.8)	0.004
range	5.75–11.06	2.29–7.03	22.3–79.3	
CT derived prostate volume (ml)				
median (mean)	46.35 (54.30)	29.56 (32.19)	29.65 (35.88)	0.005
range	31.05–90.25	16.61–65.65	22.03–55.90	
Prostate specific antigen (ng/ml)				
median (mean)	18.7 (40.9)	0.68 (0.96)	96.5 (92.9)	0.003
range	1.1–260	0.3–2.4	70.5–99.5	
	**Pretherapeutic**^1^	**Posttherapeutic**^2^	**Control group**^3^	*p*-value (*/**)
TUNEL positive cells per HPF				
median (mean)	0.6 (0.5)	3.2 (4.3)	1.35 (5.0)	0.02/0.003
range	0–1.3	2.0–8.4	0.4–3.3	
Regressive changes in % of whole tumor				
median (mean)	0	40 (42)	0	
range	0	15–80	0	

1 pretherapeutic prostate biopsy

2 prostatectomy specimen of therapy group (neoadjuvant treatment)

3 prostatectomy specimen of control group (no neoadjuvant treament)

* pretherapeutic vs. posttherapeutic

** control group vs. posttherapeutic

**Figure 1:**
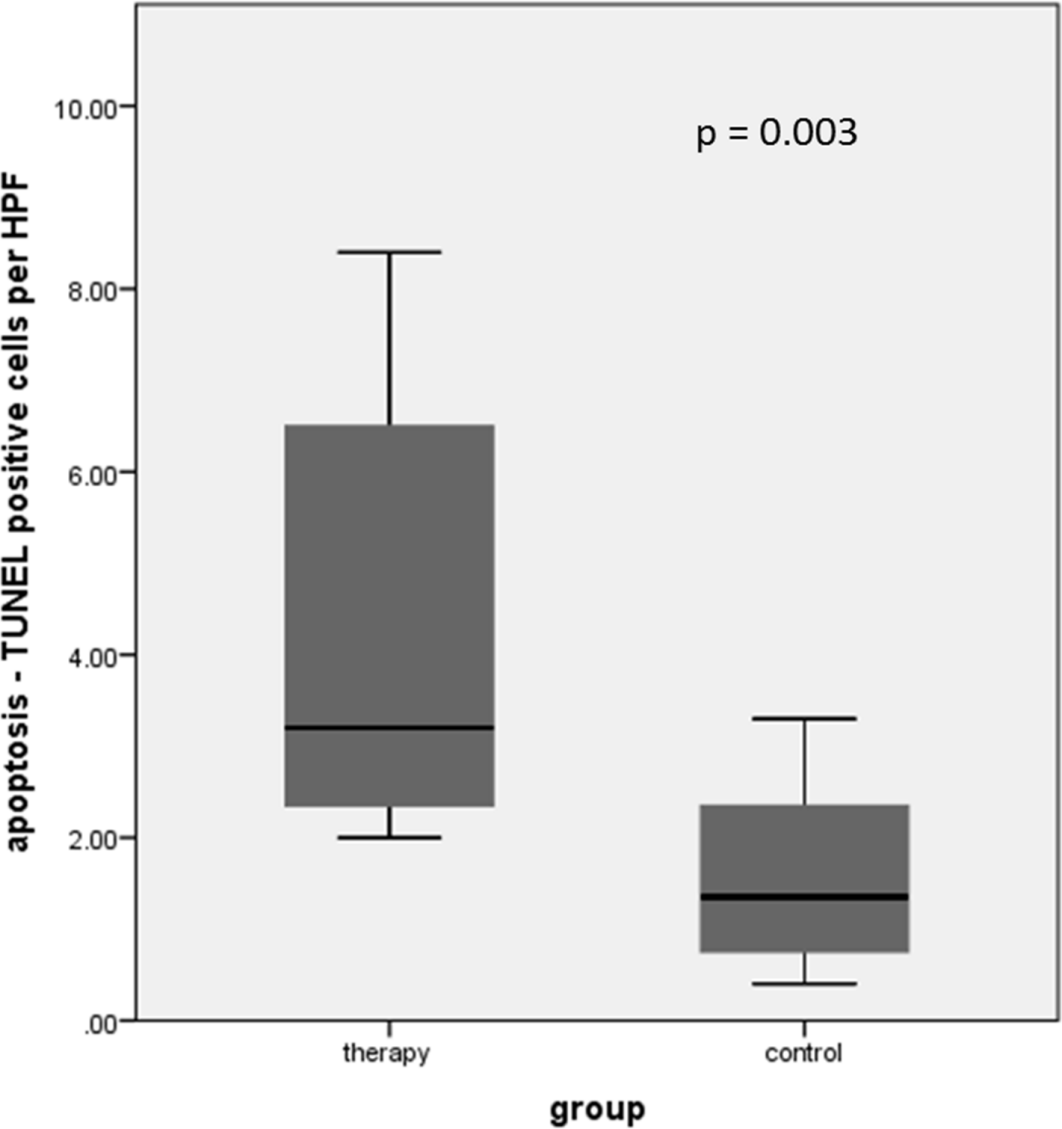
Detection of apoptosis in both patient cohorts revealed a statistically significant difference with a higher number of TUNEL positive cells per HPF in the treated patient group after neoadjuvant chemotherapy

Detection of apoptosis in pretherapeutic biopsy and prostatectomy specimens of the therapy group revealed a statistically significant difference with a higher number of TUNEL (terminal deoxynucleotidyltransferase mediated nick-end labeling) positive cells per HPF in the prostatectomy specimens (*p* = 0.02). Number of TUNEL positive cells in the prostatectomy specimen of the therapy group was statistically significantly higher compared to prostatectomy specimens of the control group without neoadjuvant therapy (*p* = 0.003) (Figure 1).

### PSA

**Figure 2:**
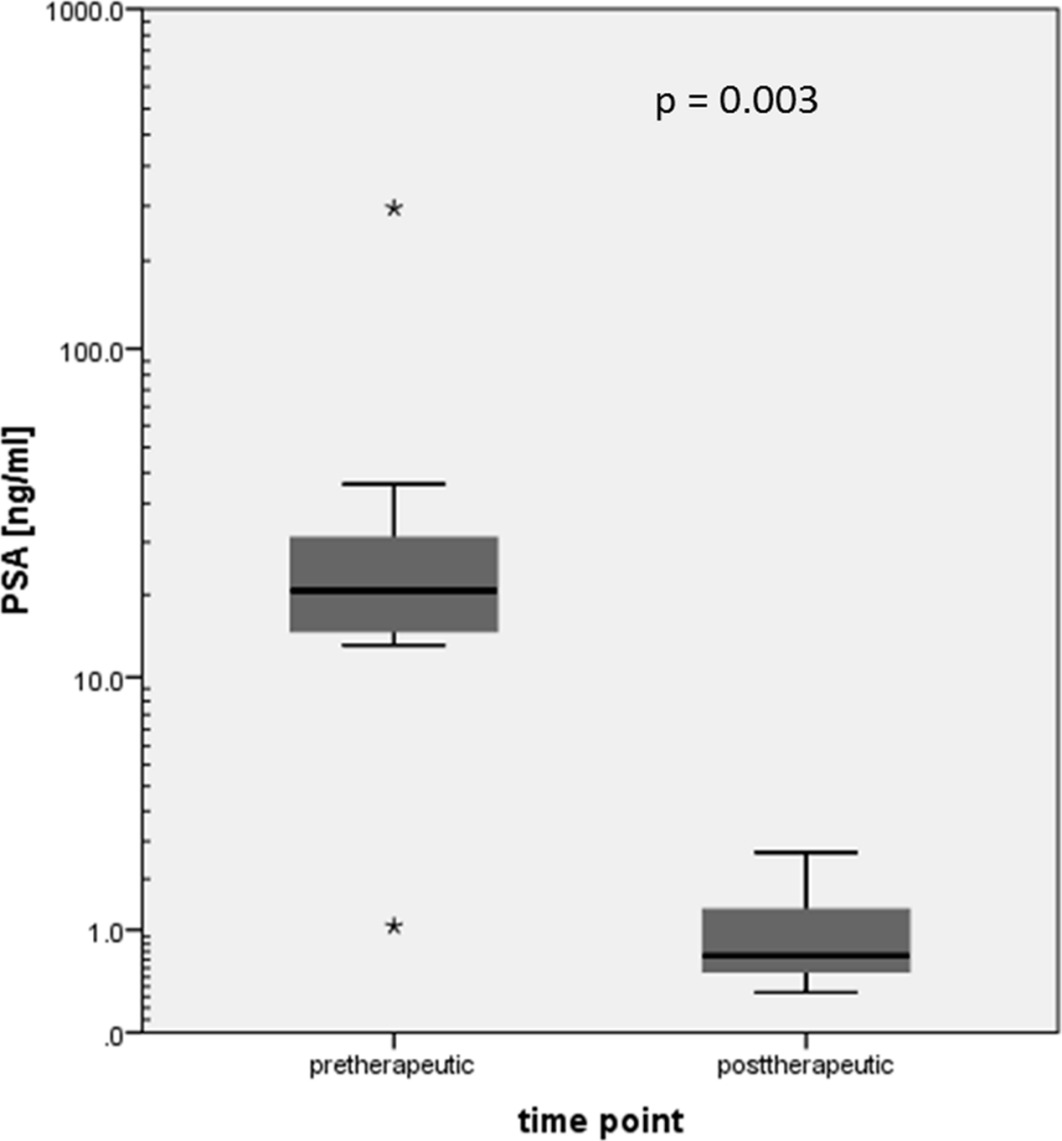
Statistically significant PSA value decrease after neoadjuvant therapy (logarithmic scale; stars indicating outliers)

Mean PSA value at the timepoint of the first [^11^C]Choline PET/CT before neoadjuvant therapy (PSA_pre_) was 40.9 ng/ml ± 73.4 (range 1.1–260). Mean PSA value after neoadjuvant therapy (PSA_post_) was 0.96 ng/ml ± 0.65 (range 0.3–2.4) (see Table 1). There was a statistically significant PSA value decrease after neoadjuvant therapy (mean decrease of 92.9 % ± 8.7 (range 70.5–99.5); *p* = 0.003) (Figure 2). The difference between PSA_post_ and PSA_pre_ (Δ PSA) was calculated for further data analysis.

### CT derived prostate volume

**Figure 3:**
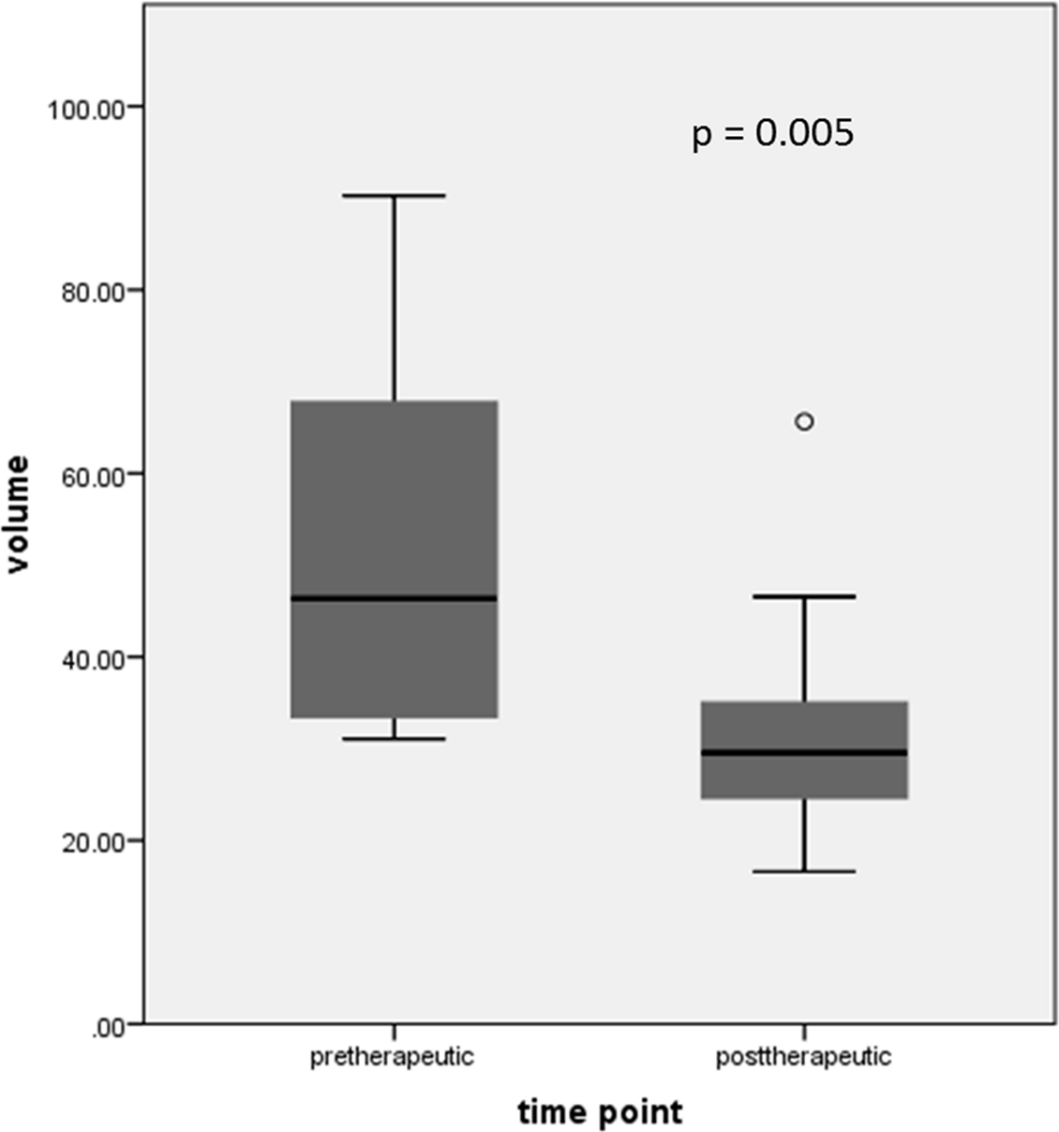
Statistically significant decrease in CT derived prostate volume after neoadjuvant therapy (dot is indicating an outlier)

Mean prostate volume before and after neoadjuvant therapy (CT prostate Vol_pre_ and CT prostate Vol_post_) was 54.3 ml ± 22.4 (range 31.1–90.3) and 32.2 ml ± 14.5 (range 16.6–65.7), respectively (see Table 1). In one patient CT prostate Vol_post_ could not be assessed due to missing CT data. There was a statistically significant decrease in CT prostate volume after neoadjuvant therapy (median decrease of 29.7 % ± 13.7 (range 22.0–55.9); *p* = 0.005) (Figure 3). The difference between CT prostate Vol_post_ and Vol_pre_ (Δ CT prostate volume) was calculated for further data analysis.

### MRI derived prostate and tumor volume

Mean prostate volume before and after neoadjuvant therapy (MRI prostate Vol_pre_ and MRI prostate Vol_post_) was 61.6 ml ± 35.7 (range 23.8–131.7) and 36.6 ml ± 16.2 (range 19.2–65.5). Mean MRI tumor volume (MRI tumor Vol_pre_ and MRI tumor Vol_post_) was 5.0 ml ± 5.4 (range 0.8– 19.1) and 3.4 ml ± 4.5 (range 0.2–15.5), respectively.

There was a statistically significant decrease in MRI prostate as well as tumor volume after neoadjuvant therapy (median decrease of 41.9% ± 13.5 (range 17.2–55.0); *p* = 0.003 and 43.5% ± 24.7 (range 5.6–75.7); *p* = 0.005, respectively). The difference between MRI prostate Vol_post_ and Vol_pre_ (Δ MRI prostate volume) as well as between MRI tumor Vol_post_ and Vol_pre_ (Δ MRI tumor volume) was calculated for further data analysis.

### [^11^C]Choline uptake

In all patients primary prostate cancer could be visualized with increased choline uptake using [^11^C]Choline PET/CT.

**Figure 4:**
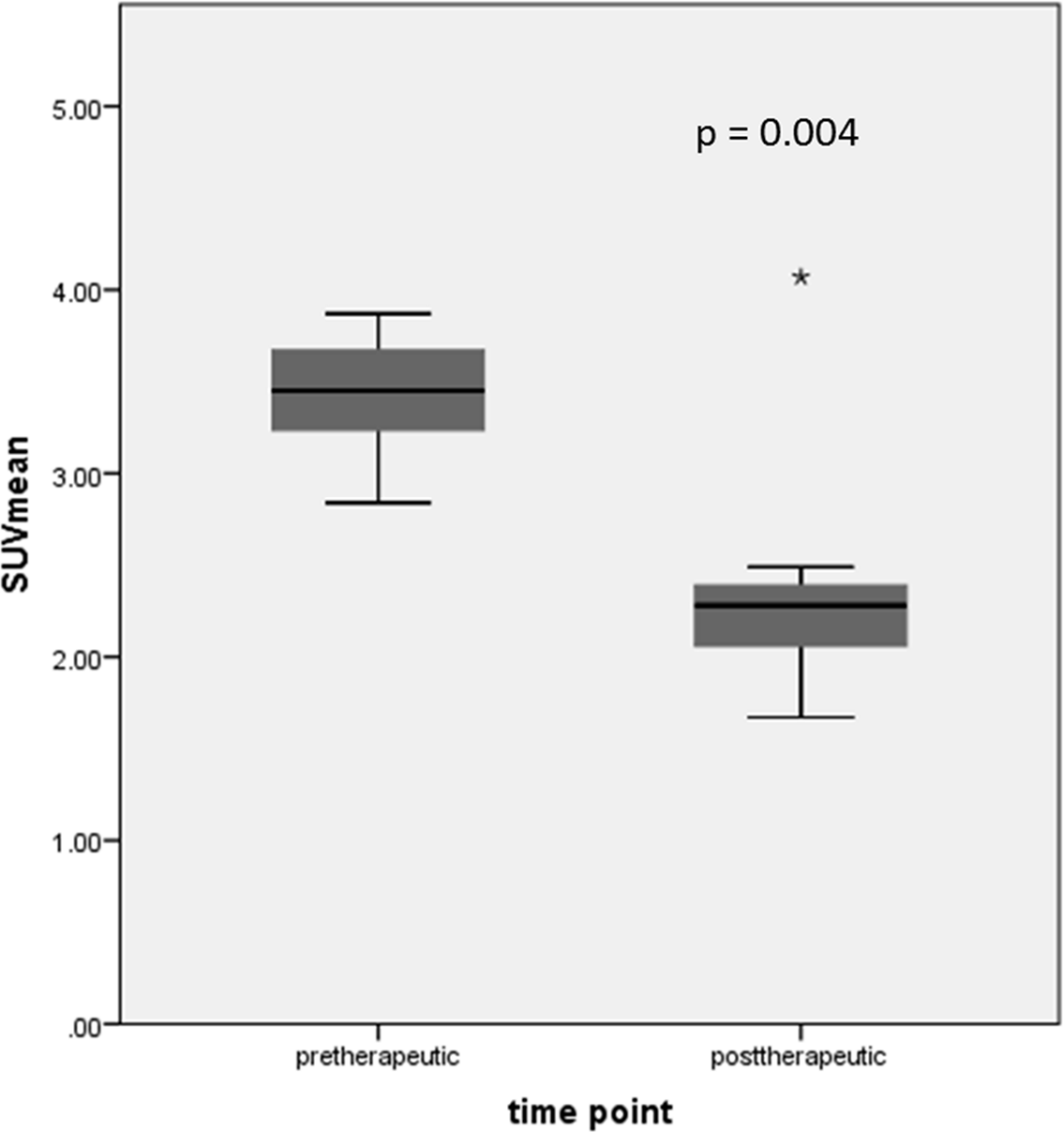
Statistically significant decrease of SUV_mean_ after neoadjuvant therapy in the whole patient group (star is indicating an outlier)

Initial mean SUV_mean_ was 3.43 ± 0.32 (range 2.84– 3.87), posttherapeutic mean SUV_mean_ was 2.36 ± 0.62 (range 1.67–4.07) (see Table 1). There was a statistically significant decrease of SUV_mean_ (30.4% ± 20.7 (range 26.4–56.9); *p* = 0.004) after neoadjuvant therapy in the whole patient group (Figure 4). In 10/11 patients a decrease in [^11^C]Choline uptake was found after neoadjuvant therapy (decrease in SUV_mean_ of 36.04% ± 8.63%). In only one patient [^11^C]Choline uptake increased after neoadjuvant therapy (initial Gleason score of 7, stage T3b, risk score 130 points); however, this patient showed a PSA decrease after neoadjuvant therapy from 1.05 ng/ml to 0.31 ng/ml. The difference of SUV_max_ as well as of SUV_mean_ (after vs. before neoadjuvant therapy) was calculated for further data analysis (Δ SUV_max_, Δ SUV_mean_).

**Figure 5:**
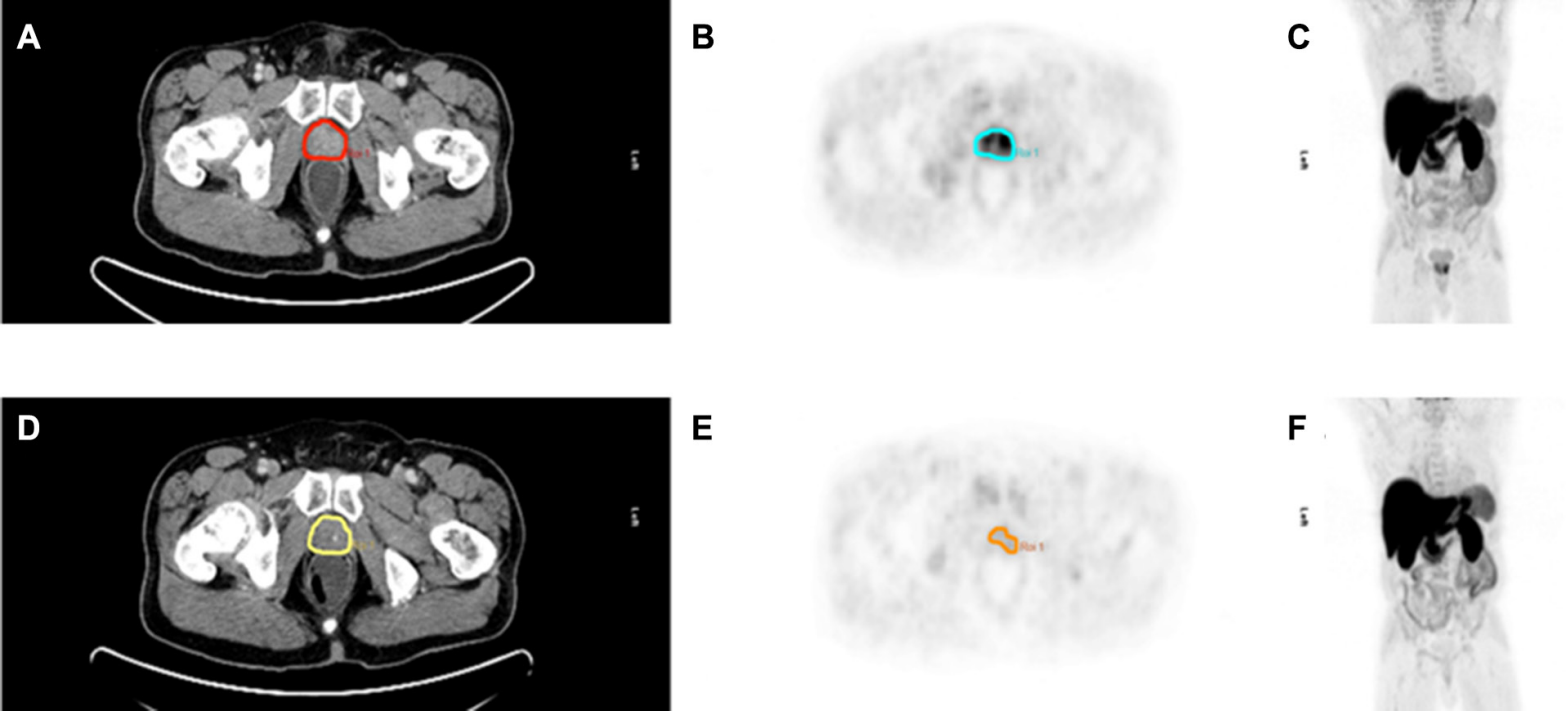
Pre- and posttherapeutic imaging example of a 66 year old patient with locally advanced prostate cancer (PSA before neoadjuvant therapy 12.63 ng/ml, Gleason score 6) before (A–C) and after neoadjuvant therapy (D–F) Significant decrease in choline uptake and prostate volume is shown in choline PET/CT after neoadjuvant therapy. PSA decreased down to 0.53 ng/ml after neoadjuvant therapy. A, B, C Pretherapeutic transaxial CT slice, PET slice, Maximum Intensity Projection (MIP) D, E, F

In the visual assessment [^11^C]Choline uptake decreased after neoadjuvant therapy in 10/11 patients. For a comparative imaging example of one patient (before and after neoadjuvant therapy) see Figure 5.

### Correlation between apoptosis (TUNEL-positive cells) and Δ PSA, Δ CT prostate volumen, Δ SUV_max_ and Δ SUV_mean_

No statistically significant correlation could be found between the number of TUNEL-positive cells per HPF in prostatectomy specimens of the therapy group and Δ PSA (r = 0.003; *p* = 0.992), Δ CT volume (r = 0.138; *p* = 0.732), as well as Δ SUV_max_ and Δ SUV_mean_ (r = 0.153; *p* = 0.673 for Δ SUV_max_ and r = 0.252; *p* = 0.483 for Δ SUV_mean_).

### Correlation of Δ SUV_max_ and Δ SUV_mean_ and Δ CT prostate volume, Δ MRI prostate and tumor volume as well as Δ PSA

There was no statistically significant correlation between Δ SUV_max_ or Δ SUV_mean_ and Δ CT prostate volumen (r = −0.062; *p* = 0.86 and r = 0.007; *p* = 0.98, respectively), Δ MRI prostate volumen (r = −0.091; *p* = 0.80 and r = −0.195; *p* = 0.59, respectively), Δ MRI tumor volumen (r = −0.127; *p* = 0.73 and r = −0.541; *p* = 0.12, respectively) as well as Δ PSA (r = 0.23; *p* = 0.49 and r = 0.208, *p* = 0.538).

## DISCUSSION

The results of this study show that [^11^C]Choline PET/CT might be useful in monitoring neoadjuvant therapy in locally advanced and high risk prostate cancer patients. Neoadjuvant therapy led to a significant decrease in choline uptake in 10/11 patients (mean decrease in SUV_mean_ of 36.04% ± 8.63%). This decrease in choline uptake in the therapy group was paralleled by regressive and apoptotic changes in histopathology. In neoadjuvantly treated patients, prostatectomy specimens showed an average of 40% therapy associated regressive changes, whereas in pretherapeutic biopsy specimens of the therapy group as well as in prostatectomy specimens of the control group no regressive changes could be shown. Compared to pretherapeutic biopsy specimens as well as to the control group, a statistically significantly higher number of TUNEL positive cells was found after neoadjuvant chemotherapy (*p* = 0.02 and 0.003, respectively).

Synergistic therapeutic effects of docetaxel chemotherapy and complete androgen blockade may contribute to these effects. Docetaxel increases apoptosis via inhibition of depolymerisation of microtubules and genetic modifications such as induction of expression of apoptosis related genes in prostate cancer cells [19]. Also withdrawal of androgens leads to apoptosis of prostate cancer cells as described by Floyd et al. 2009 [20].

To our knowledge, there are no human studies in which both modulation of choline signal as well as histopathological assessment of therapy associated regressive changes and apoptosis were evaluated. In a preclinical study Davoodpour et al. 2004. showed a statistically significant increase in apoptotic nuclei in PC-3 prostate cancer cell aggregates following 2-methoxyestradiol therapy; however, the uptake of labeled choline markers failed to record the growth inhibitory effects of 2-methoxyestradiol PC-3 cell aggregates [21]. Tang et al. 2006 [22] showed an increase of apoptosis after neoadjuvant docetaxel therapy in LNCaP prostate cancer xenograft mouse tumors. TUNEL assays confirmed a significantly greater density of apoptotic tumor cells after docetaxel therapy followed by castration compared to castration only. In this study no metabolic imaging was performed.

In our study apoptotic changes were paralleled by a significant decrease in [^11^C]Choline uptake after combined neoadjuvant therapy. Accordingly, in preclinical studies with PC-3 and LNCaP prostate cancer xenograft mouse models a decrease in [^11^C]Choline tumor uptake could be observed as early as one week after start of docetaxel therapy [23, 24]. This reduction of [^11^C]Choline uptake is most likely due to a docetaxel-induced increase of cell apoptosis which leads to cell death in a large proportion of tumor cells resulting in a reduced number of surviving choline positive tumor cells.

In accordance to our results, other human studies have shown that antiandrogen therapy leads to a modulation of the [^11^C]Choline signal in prostate cancer tumors. Fucchio et al. 2011 showed that androgen deprivation therapy significantly reduced [^11^C]Choline uptake in androgen sensitive prostate cancer patients [25]. Giovacchini et al. 2008 [18] showed a significant reduction of [^11^C]Choline uptake in 6 patients with biopsy-proven prostate cancer before and after bicalutamide therapy (150 mg/d, mean therapy duration of 4 months) (SUV_max_ of 11.8 ± 5.3 and 6.4 ± 4.6, respectively; percentage decrease of 45% ± 32). These data are in accordance with the results by Challapalli et al. 2014 [17] showing a decrease of [^11^C]Choline uptake after goserelin (LHRH analoga) therapy (mean duration of 77 days) in 10 patients. The authors evaluated patients with localized prostate cancer, whereas in our study patients with locally advanced and high risk prostate cancer were included. However, initial [^11^C]Choline uptake before antiandrogen therapy was comparable to our data. Mean decrease in [^11^C]Choline uptake (SUV_max_) after neoadjuvant therapy was 39%, comparable to the results of our study with a SUV_max_ decrease of 47.8 % ± 25.8.

Giovacchini et al. 2008 [18] stated that a thinning and atrophy of glandular cells, a reduction of choline transporter or choline kinase activity and - as shown by Swinnen et al. 1998 [26] - a reduction in expression of genes involved in the lipid metabolism might be reasons for the decrease of choline uptake after bicalutamide therapy.

In the studies discussed above no histopathological assessment of apoptosis was performed; therefore, correlation between choline signal modulation and apoptotic rate could not be evaluated. The results of our study showed no significant correlation between decrease in [^11^C]Choline uptake and number of apoptotic nuclei in posttherapeutic prostatectomy specimens of the therapy group. This might be due to the small patient cohort. However, to the best of our knowledge we are the first to analyse the association of therapy induced apoptosis and change of choline uptake in prostate cancer. Comparing our data with those of Giovacchini et al. 2008 [18] and Challapalli et al. 2014 [17] it has to be taken into account that in our study a combination of complete androgen blockade and docetaxel chemotherapy was applied. Therefore both the effects of androgen deprivation therapy as well as docetaxel treatment contribute to the decrease of [^11^C]Choline uptake in our study. To our knowledge, there are no studies reporting on the change in [^11^C]Choline uptake after combined neoadjuvant therapy.

Besides a reduction of [^11^C]Choline uptake we found a statistically significant prostate and tumor volume reduction. In the phase II study by Thalgott et al. 2014 [11] (of which our patient group is a subgroup) MRI showed significant down staging, median prostate and tumor volume reduction was 37.1% and 46.4%, respectively. In our subgroup significant median prostate and tumor volume reduction was similar (41.9% and 43.5%, respectively). Tumor volume reduction or tumor growth inhibition following chemotherapy was also shown in previous preclinical studies [23, 19, 24, 22].

In our study there was no significant correlation between decrease in [^11^C]Choline uptake and prostate and tumor volume reduction assessed by CT and MRI. That means that - besides morphological information provided by CT and MRI - [^11^C]Choline PET/CT offers additional independent information on metabolic response after combined neoadjuvant therapy.

### PSA

In our subgroup PSA value statistically significantly decreased after combined neoadjuvant therapy (PSA decrease of 92.9% (range 70.5–99.5)). Thalgott et al. 2014 [11] found a similar PSA reduction of 97.3% (range 81.3–99.9) in the whole patient group.

Giovacchini et al. 2008 [18] showed a statistically significant PSA reduction of 78% after neoadjuvant bicalutamide therapy. The decrease in PSA values was higher compared to the decrease of SUV_max_. Challapalli et al. 2014 [17] found a mean PSA decrease after neoadjuvant goserelin therapy of 94%. These results are in line with our results showing a mean PSA decrease of 92.9%. Additionally, Challapalli et al. 2014 [17] showed a significant correlation between PSA reduction and the decrease of [^11^C]Choline uptake (SUV_mean_) (r = 0.7; *p* = 0.04), but no correlation with decrease in SUV_max_. Similarly, in our study no correlation could be shown between PSA reduction and decrease in [^11^C]Choline uptake.

### Limitations

### Patient population

The patients included in this study belonged to a highly selective subgroup of the phase II study by Thalgott et al. 2014 [11], the patient sample was rather small. This is also a limitation of other studies, Giovacchini et al. 2008 [18] included only six patients, Challapalli et al. 2014 [17] 10 patients. As a further limitation in four of the patients not all imaging and immunohistochemistry data were available. The study population of the control group (additionally used for comparative assessment of apoptosis) was matchpaired to the therapy group; however control group showed a significantly different age compared to the therapy group.

### Methodical limitations

All VOIs (in pre- and post-therapy PET and CT images) have been placed manually causing an intra-observer variability.

Due to the study design, all patients received combined neoadjuvant therapy (docetaxel plus bicalutamide and buserelin), therefore only synergistic effects of this combined therapy can be monitored - single effects on [^11^C]Choline uptake cannot be separated/differentiated in this study. As [^11^C]Choline PET/CT was performed at the end of neoadjuvant treatment, the results did not influence further treatment decisions.

## MATERIALS AND METHODS

Our patient cohort is a subgroup of the study cohort of a phase II study by Thalgott et al. 2014 [11] who used sole MRI for therapy response assessment.

Our subgroup included 11 patients (mean age of 68, 72 ± 5, 83, range: 52–76 years) with biopsy-proven locally advanced and high risk prostate cancer with a mean biochemical recurrence risk of 79.3% within 5 years (range 45%–95%) according to Kattan's preoperative nomogram (risk score ≥ 120 points (mean 162, range 125–200)) [3, 11] who underwent [^11^C]Choline PET/CT before and after combined neoadjuvant docetaxel chemotherapy and complete androgen blockade before RPE and pelvic lymph node dissection (LND). For detailed patient characteristics see Table 2.

**Table 2:** Summary of therapy group and control group patient characteristics

Clinical characteristics	Therapy group	Control group	*p*-value
Patients (*n*)	11	11	
Age (yr.)			0.023
median (mean)	70 (69)	65 (66)	
range	52–76	60–72	
Prostate specific antigen (ng/ml)			0.365
median (mean)	18.7 (40.9)	13.8 (17)	
range	1.1–260	3.7–34	
Gleason score at diagnosis			0.171
6	2 (18%)	1 (9%)	
7	5 (46%)	3 (27%)	
8	2 (18%)	2 (18%)	
9	2 (18%)	4 (37%)	
10	0 (0%)	1 (9%)	
Clinical stage			0.519
T2c	1 (9%)	5 (45%)	
T3a	4 (36%)	0 (0%)	
T3b	6 (55%)	6 (55%)	

Inclusion criteria were absence of bone metastases (determined by bone scan) and lymph node metastases (in diagnostic contrast-enhanced CT and [^11^C]Choline PET/CT), life expectancy > 10 years and sufficient hematological, renal and hepatic function.

Exclusion criteria were a former cytotoxic chemotherapy or androgen deprivation therapy, radiation treatment, cryotherapy or high intensive focused ultrasound, known second malignancy (with the exception of curatively treated non-melanotic skin tumors), peripheral neuropathy > grade II, contraindication against steroidal therapy, active infections and other medical relevant adverse conditions [11].

This study is a prospective analysis of therapy response monitoring using [^11^C]Choline PET/CT in a subgroup of a phase II trial that was conducted at the Department of Urology, Technische Universität München [11]. The study was approved by the institutional review board and was performed in accordance with the ethical standards of the Declaration of Helsinki. All patients gave written informed consent.

For assessment of apoptotic cells the TUNEL method was used. The number of apoptotic cells was assessed in the pretherapeutic biopsy specimens and posttherapeutic prostatectomy specimens of the study cohort and additionally in prostatectomy specimens of a control group (without neoadjuvant treatment) with matchpaired patients, who presented with locally advanced and high risk prostate cancer and received a primary prostatectomy without prior neoadjuvant therapy. Statistical analysis (Mann-Whitney-*U* test for unpaired samples) confirmed no significant difference of therapy and control group with respect to T stage, Gleason score and PSA value; however, age was significantly different between both groups (for patient characteristics and *p*-values see Table 2).

### Treatment plan

All patients received a combined therapy of docetaxel and complete androgen blockade (docetaxel 75 mg/m^2^ body surface i.v. over 60 minutes on day 8, 29 and 50; antiandrogen therapy with bicalutamide (Casodex^®^) (50 mg per os daily beginning at day one until the day of surgery) as well as buserelin (3-month depot preparation, singular subcutaneous injection on day 3 (9.45 mg)).

For further information on concomitant dexamethasone/prednisolone premedication see Thalgott et al. 2014 [11]. Afterwards RPE and pelvic LND were performed between day 64 and 78.

### [^11^C]Choline PET/CT imaging protocol

[^11^C]Choline PET/CT imaging was performed before start of therapy and between 7–21 days (mean 15 days) after neoadjuvant therapy. Patients fasted at least 6 hours before [^11^C]Choline PET/CT scanning. Patients underwent [^11^C]Choline PET/CT (midthigh to thorax) (Sensation 16 Biograph PET-CT-Scanner (Siemens Medical Solutions) (ACCEL PET FWHM = 6.5 mm) five minutes after injection of 802 ± 23.6 MBq and 775 ± 50.8 MBq of [^11^C]Choline before and after neoadjuvant therapy, respectively. The acquisition protocol included a low-dose CT (26 mA, 120 kV, 0.5 seconds per rotation, 5-mm slice thickness) for attenuation correction, followed by the PET scan and a diagnostic CT in portal venous phase 80 seconds after i.v. injection of contrast agent (Imeron 300; 240 mA, 120 kV, 0.5 seconds per rotation, 5-mm slice thickness). Following the standard protocol, all patients received a rectal filling with a negative contrast agent (100–150 mL). All PET scans were acquired in 3-dimensional (3D) mode with an acquisition time of 3 minutes per bed position. Image reconstructed was carried out iteratively by an ordered subsets expectation maximization algorithm (4 iterations, 8 subsets) followed by a post-reconstruction smoothing Gaussian filter (5 mm FWHM).

### Synthesis of [^11^C]Choline

[^11^C]Choline was synthesized according to the method of Pascali et al. 2000 [27] with minor modifications. [^11^C]Choline was produced with radiochemical yields of 80% to 90%, based on [^11^C]Methyl Iodide, and radiochemical purity of > 99%.

### [^11^C]Choline PET/CT image analysis

Images were analyzed by two experienced nuclearmedicine physicians.

For image analysis the transaxial PET slices and the corresponding fused low-dose CT slices were used. We evaluated [^11^C]Choline uptake in the prostate semiquantitatively using SUV_max_ and SUV_mean_ (applying volumes of interest (VOI) with a threshold of 50%) derived from attenuation–corrected PET emission data. For delineation of prostate borders and assessment of prostate volume a CT derived VOI was used.

### MRI imaging protocol and image analysis

MRI imaging of the prostate was performed with an endorectal coil (1.5 T, Magnetom Avanto, Siemens, Germany) before and after neoadjuvant therapy. T2-weighted sequences in three planes, axial T1-weighted and a dynamic-contrast-enhanced sequence was acquired and analyzed independently by a board certified radiologist. Prostate and tumor volumes were calculated, tumor volume was determined by measuring the maximum tumor area in the axial plane and the longest cranio-caudal diameter (for further details see [11]).

### Histopathological analysis and assessment of apoptosis

After prostatectomy, tissues were fixed in 10% neutral-buffered formalin, cut into slices of 0.5 cm thickness and embedded in paraffin. Three μm whole-mount sections were cut, mounted on slides and stained with haematoxylin and eosin (H.-E.). The percentage of tumor tissue showing therapy-associated regressive changes was estimated according to Srigley et al. 2012 [28]. Representative tumor regions regarding growth patterns, grading and tumor regression were selected of each specimen and stained with the Apoptag^®^ Plus Peroxidase *in situ* detection kit (Millipore, S7101) to determine the apoptotic rate according to the TUNEL method using diaminobenzidine (DAB) as detection system. Counterstaining was performed with haematoxylin. Stained sections were visualized under bright field microscope and the number of TUNEL positive tumor cells per high power field (HPF) was determined as the average of at least 10 HPFs (prostatectomy specimen of the therapy and control group) or 5 HPFs (pretherapeutic biopsies of the therapy group), respectively.

### Statistical analysis

Statistics were performed using Microsoft Excel 2008 and SPSS (version 20.0).

Kolmogorov-Smirnov test revealed no normal distribution of data; therefore, statistics were performed using non-parametric tests. Wilcoxon signed-rank test for paired samples was applied to test for significance in changes of choline uptake (SUV_max_ and SUV_mean_), reduction of prostate and tumor volume and change in PSA values (before and after neoadjuvant therapy). Wilcoxon signed-rank test for paired samples was also used to test for differences in apoptosis (as number of TUNEL-positive cells per HPF) between pretherapeutic biopsy and posttherapeutic prostatectomy specimens of the therapy group. Mann-Whitney-*U* test for unpaired samples was used to test for differences in apoptosis between prostatectomy specimens of the therapy group and control group (without neoadjuvant therapy). Correlations between these single parameters (change in choline uptake, PSA, prostate and tumor volume as well as apoptosis) were tested using SPSS.

## CONCLUSIONS

[^11^C]Choline PET/CT imaging showing a modulation of [^11^C]Choline uptake can be useful in monitoring combined neoadjuvant therapy in patients with locally advanced and high risk prostate cancer. Decrease in imaging biomarker signal is paralleled by an increase of apoptosis. Further studies are recommended to evaluate the potential of [^11^C]Choline PET/CT during the course of neoadjuvant therapy for early response assessment, possibly having impact on further treatment decisions.
